# Possible Roles of Strigolactones during Leaf Senescence

**DOI:** 10.3390/plants4030664

**Published:** 2015-09-11

**Authors:** Yusuke Yamada, Mikihisa Umehara

**Affiliations:** Graduate School of Life Sciences, Toyo University, 1-1-1 Izumino, Itakura-machi, Ora-gun, Gumma 374-0193, Japan; E-Mail: dwarf1012y.y@gmail.com

**Keywords:** leaf senescence, phytohormones, nutrient deficiency, strigolactone, translocation

## Abstract

Leaf senescence is a complicated developmental process that involves degenerative changes and nutrient recycling. The progress of leaf senescence is controlled by various environmental cues and plant hormones, including ethylene, jasmonic acid, salicylic acid, abscisic acid, cytokinins, and strigolactones. The production of strigolactones is induced in response to nitrogen and phosphorous deficiency. Strigolactones also accelerate leaf senescence and regulate shoot branching and root architecture. Leaf senescence is actively promoted in a nutrient-poor soil environment, and nutrients are transported from old leaves to young tissues and seeds. Strigolactones might act as important signals in response to nutrient levels in the rhizosphere. In this review, we discuss the possible roles of strigolactones during leaf senescence.

## 1. Introduction

Leaf senescence is an essential process observed in the final stage of leaf development [1]. In particular, age-dependent senescence in monocarpic plants precedes whole plant death at the end of its life cycle. During leaf development, leaf cells accumulate ribulose bisphosphate carboxylase (rubisco), chlorophyll-*a/b* binding protein (CAB), and other proteins involved in photosynthesis in the chloroplast, which contains more than 70% of the total leaf protein [2]. Photosynthesis fixes carbon using light energy and plays a central role in plant growth. When photosynthetic activity decreases in the lower (old) leaves, these leaves are no longer required and nutrients accumulated in them are recycled. In the early stage of leaf senescence, cellular metabolism and gene expression patterns change dramatically. The most important change is chloroplast breakdown, which is accompanied by chlorophyll degradation and progressive loss of chloroplast proteins, such as rubisco and CAB, and which causes leaf color changes from green to yellow. After chloroplast degeneration, plasma membrane integrity is lost as the final step of cell death, and cellular ionic substances leak out [3,4]. Some senescence-associated genes (SAGs) encode enzymes involved in protein, lipid, and nucleic acid degradation [5]. However, leaf senescence is not a passive and unregulated degeneration process. The degraded cellular compounds and minerals are transported out of the senescing leaf and back into the main plant body, where carbon, nitrogen, and mineral nutrients are redistributed to the growing parts, especially to young organs and seeds, and reused for synthetic processes [2].

Leaf senescence is not only controlled by the developmental stage but is also influenced by various internal and environmental cues, even in young plants. Major environmental cues include light intensity, temperature, drought, pathogen attack, and soil nutrient deficiency [2]. Plant hormones act as internal cues that influence plant development and responses to environmental stresses, and play an important role in the regulation of leaf senescence. In general, ethylene, jasmonic acid, and salicylic acid are involved in plant immune responses, such as responses to pathogen attacks and in wounding, and abscisic acid mediates plant osmotic response [6,7]. Cytokinins control nutrient remobilization between source and sink organs. Ethylene, jasmonic acid, salicylic acid, and abscisic acid act as positive regulators in leaf senescence, whereas cytokinins are potent inhibitors of leaf senescence [7]. In addition to these hormones, strigolactones (SLs, a class of plant hormones) appear to regulate leaf senescence because some SL-deficient and SL-insensitive mutants show delayed leaf senescence [8,9,10,11]. In this review, we discuss the physiological roles of SLs in leaf senescence.

## 2. SL Pathway

**Figure 1:**
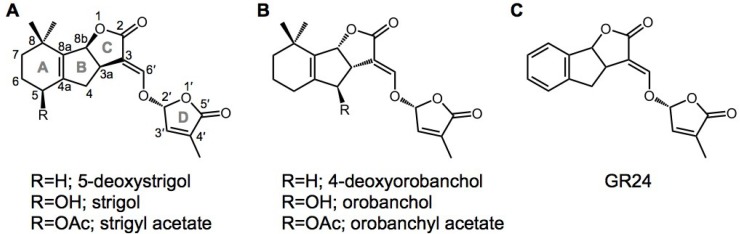
Chemical structures of strigolactones (SLs) and the karrikin (KAR). Natural SLs of (**A**) the strigol type and (**B**) the orobanchol type are shown as representative SLs that have been identified from root exudates of various plant species; (**C**) the synthetic SL analog GR24 is widely used to evaluate the effects of SLs.

SLs are a group of terpenoid lactones that consist of a tricyclic lactone (ABC-ring) and hydroxymethyl butenolide (Figure 1). SLs were originally identified as seed germination stimulants in root parasitic plants such as *Striga hermontica* [12,13]. In 2005, SLs were characterized as inducers of hyphal branching in arbuscular mycorrhizal fungi [14]. Although SLs were well known as communication signals for parasitic and symbiotic interactions, they were later rediscovered as phytohormones for plant growth. Shoot branching inhibition by SLs was the first such discovery [15,16]. A series of mutants with enhanced shoot branching were isolated, including *ramosus* (*rms*) in pea, *decreased apical meristem* (*dad*) in petunia, *more axillary growth* (*max*) in Arabidopsis, and *dwarf* (*d*)/*high tillering dwarf* (*htd*) in rice. Grafting experiments with these mutants suggested that a root-derived graft-transmissible signal is involved in shoot branching inhibition [17]. Several *RMS*/*DAD*/*MAX*/*D* genes, mutations which cause the enhanced shoot branching phenotype, have been identified by molecular cloning. The genes *MAX3*/*RMS5*/*D17*/*DAD3* and *MAX4*/*RMS1*/*DAD1*/*D10* encode carotenoid cleavage dioxygenases 7 (CCD7) [18,19,20,21] and 8 (CCD8) [8,22,23,24], respectively, suggesting that the branch-inhibiting signal is derived from a carotenoid. MAX1 is a member of the cytochrome P450 superfamily, and acts downstream of CCD7 and CCD8 [25]. MAX2/RMS4/D3 is an F-box protein [21,26,27] and is the substrate-recognition subunit in the SKP1-CUL1-F-box-protein (SCF) ubiquitin E3 ligase complex, which targets proteins for proteasomal degradation [28]. Some F-box proteins are involved in the perception of plant hormones such as auxins, gibberellins, and jasmonic acid [28]. Endogenous SL levels are very low in *ccd7*, *ccd8*, and *max1* mutants, and exogenously applied SLs inhibit enhanced shoot branching in these mutants, whereas the *max2*/*rms4*/*d3* mutant is insensitive to SL treatment [15,16].

**Figure 2:**
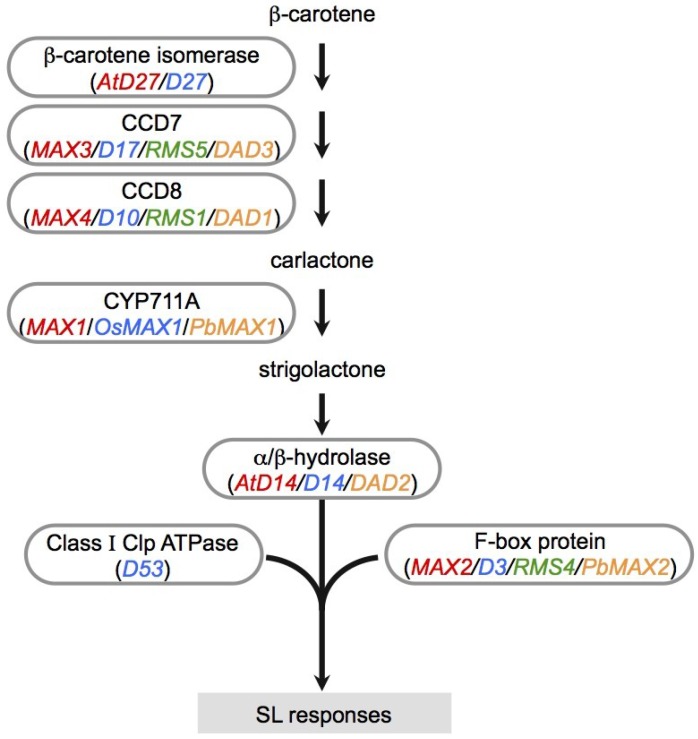
SL biosynthesis and signaling pathway. The SL precursor carlactone is produced from β-carotene by three enzymes, β-carotene isomerase, CCD7, and CCD8. MAX1 is CYP711A, a member of the cytochrome P450 family, and acts as an oxidase for conversion of CL to SL. D14 (α/β-hydrolase) and F-box protein form an SCF-type ubiquitin ligase complex required for SL signaling. D53 is targeted for degradation by the SCF complex in SL signaling. Gene color coding: red, Arabidopsis; blue, rice; green, pea; orange, petunia.

Later, another SL biosynthesis gene, *D27*, was identified in rice and Arabidopsis. *D27* encodes an iron-containing protein localized in plastids; *D27* does not have any conserved domains or homology to any known enzymes [29,30]. Recently, D27 was shown to be a β-carotene isomerase, and carlactone (CL), an SL-like carbon skeleton, was synthesized from β-carotene by three biosynthetic enzymes, D27, CCD7, and CCD8 *in vitro* (Figure 2) [31]. Seto *et al.* reported that endogenous CL was detectable in rice and Arabidopsis, and that ^13^C-labeled CL was converted to ^13^C-labeled SL, suggesting that CL is a biosynthetic precursor of SLs [32]. MAX1 catalyzes CL conversion to carlactonic acid upstream of SLs [33].

In SL signaling, *AtD14*/*DAD2*/*D14* and *D53* function downstream of *MAX1* as signaling components, as well as *MAX2*/*RMS4*/*D3* (Figure 2). D14 belongs to the α/β-hydrolase family [34]. Crystal structure analysis of D14 and its orthologs from petunia (DAD2) and Arabidopsis (AtD14) demonstrated that the catalytic triad (Ser, His, Asp) is structurally conserved in these species [35,36,37]. Recently, a dominant SL-insensitive rice mutant, *d53*, was characterized [38,39]. The D53 protein interacts with D14, which binds GR24 (a synthetic SL analog; Figure 1) and is degraded through the D3-dependent proteasome pathway; D53 is thought to be a repressor of signaling downstream of SL [38,39]. Recently, it was reported that SLs control not only shoot branching, but also secondary growth, leaf senescence, and root growth [40,41,42,43,44,45].

## 3. Leaf Senescence Is Influenced by SL Signaling

Mutants with delayed leaf senescence in the dark were screened by using Arabidopsis seeds mutagenized with ethylmethane sulfonate or by T-DNA insertion, and some mutants were named *oresara* (*ore*, oresara means “long living” in Korean) [46]. Among the *ore* mutants, *ore9* shows excessive shoot branching and delayed leaf senescence; *ORE9* was later shown to be identical to *MAX2* [9,27]. *D3* is a *MAX2*/*ORE9* ortholog in rice; the *d3* mutant also shows delayed leaf senescence [10]. Not only the SL-insensitive mutants *max2* and *d3* but also SL biosynthesis mutants exhibit the delayed senescence phenotype in petunia and *Lotus japonicus* [8,47]. Not all SL mutants, however, show the delayed leaf senescence. No altered senescence phenotype has been reported for SL mutants/transgenics in pea or tomato [48,49,50]. To explore the effects of SLs on leaf senescence, GR24 was applied to leaf segments of rice and Arabidopsis incubated in the dark [44,45]. Leaf yellowing and membrane ion leakage from leaf segments were measured in the SL mutants as indicators of leaf senescence. Exogenously applied GR24 accelerated leaf senescence in the SL-deficient mutants *d27*, *d17*, and *d10* of rice and *max1*, *max3*, and *max4* of Arabidopsis, but appeared to have no effect on the SL-insensitive mutants *d3* and *d14* of rice and *max2* and *atd14* of Arabidopsis. SAGs are used as marker genes that are upregulated during both dark-induced and natural leaf senescence [51]. Among the SAGs, Yamada *et al.* [44] used the genes for α-keto acid dehydrogenase, which is a key enzyme in the catabolic pathway for branched-chain amino acids in mitochondria [52]; isocitrate lyase, which cleaves isocitrate to produce glyoxylate in the glyoxylate cycle [53]; and aspartic protease, which has been implicated in protein processing or degradation during plant senescence [54]. Although there were no significant differences in color, chlorophyll levels, or ion leakage between GR24 treatment and the control in wild-type leaves, SAG expression levels increased in wild-type plants and SL-deficient mutants after GR24 treatment, indicating that GR24 induces SAGs during senescence [44]. In senescing and fully senescent leaves, the transcript levels of SL biosynthesis-related genes *MAX1*, *MAX3*, and *MAX4* are upregulated, suggesting that they play a role in leaf senescence [45]. SL levels in leaves are still unknown because they are too low to be measured. SLs might be produced in senescing leaves but not in young leaves. Carlactonic acid or methyl carlatonate, recently found SL-like compounds, might be synthesized in leaves and control the senescence [33]. It remains unknown whether SLs are synthesized in the leaf during senescence or SLs transported from roots promote leaf senescence.

Ethylene promotes leaf senescence. In Arabidopsis, five ethylene receptors, ETHYLENE RESISTANT1 (ETR1), ETR2, ETHYLENE INSENSITIVE4 (EIN4), ETHYLENE RESPONSE SENSOR1 (ERS1), and ERS2, have been identified; these receptors activate the serine/threonine kinase CTR1, a negative regulator of ethylene signaling [55]. Ethylene binding to the receptors inactivates CTR1, resulting in the translocation of the C-terminal fragment of the positive regulator EIN2 to the nucleus to stabilize the transcription factors EIN3 and ETHYLENE INSENSITIVE-LIKE1 (EIL1) [56]. EIN3 and EIL1 activate the expression of ethylene target genes. The ethylene-insensitive mutants *ein2* and *ein3 eil1* exhibit a strong delayed leaf senescence as well as SL mutants in the dark [45]. Both GR24 and ethylene treatments strongly promote leaf senescence in comparison with only ethylene treatment. These results suggest that SLs promote leaf senescence by enhancing the effects of ethylene. However, *max1 ein2* double mutants show a stronger delayed leaf senescence phenotype than *max1* or *ein2* single mutants, suggesting that SLs partially promote leaf senescence through an ethylene-independent pathway.

In contrast, cytokinins inhibit leaf senescence. In Arabidopsis, the *ahk2 ahk3* double mutants, which have defective cytokinin receptors, did not display the inhibition of dark-induced leaf senescence observed in wild type by exogenously applied cytokinin [57]. Delayed leaf senescence was shown in *ore12*, which has a recessive missense mutation in *AHK3* [58]. In some plant species, SL mutants show delayed leaf senescence. However, the cytokinin level in xylem sap was rather reduced in SL mutants of Arabidopsis and pea, implying feedback regulation of xylem sap cytokinins [48,59,60]. In shoot branching regulation, strigolactones and cytokinins are thought to be transported acropetally through the xylem and act directly to control axillary bud outgrowth through joint regulation of a TCP (for TB1, CYCLOIDEA, PCF domain) transcription factor BRC1 [61,62]. In Arabidopsis, when wild-type and SL-deficient mutant *max4* was grafted as scion and stock, respectively, leaves of wild type exhibited early senescence compared to that of *max4* [45]. The main SLs to promote leaf senescence might be synthesized in leaves because *max4* cannot produce SLs. To explore the interaction and transport further, it would be necessary to measure cytokinins and small amounts of SLs in leaves during senescence.

MAX2 is involved not only in SL, but also in karrikin (KAR) signaling in Arabidopsis [63,64]. KARs are a class of butenolide compounds; they promote seed germination and repress hypocotyl elongation. KARRIKIN INSENSITIVE2 (KAI2) is the AtD14 paralog required for KAR signaling [64]. KAI2 transduces the hormone signals to the SCF complex containing F-box protein MAX2 [65]. SUPPRESSOR OF MAX2 1 (SMAX1) is a possible suppressor in KAR signaling [66]. In dark-induced leaf senescence of *kai2-3* and *smax1*, a delayed-senescence phenotype was not observed, suggesting that SL signaling specifically regulates leaf senescence but not KAR signaling [45,66].

## 4. Regulation of Leaf Senescence by SLs under Phosphate Deficiency

In various plants, SL levels are increased in response to the deficiency of nutrients such as nitrogen and phosphate (Pi) [16,67,68,69,70,71]. Conversely, nitrogen and Pi fertilization rapidly suppress SL production [72]. Pi is an important structural component of nucleic acids and membrane lipids and is also required for regulatory pathways involving phospholipid-derived signaling molecules or protein phosphorylation [73]. Thus, plant growth is generally suppressed by Pi deficiency. In rice, Pi deficiency was found to reduce shoot and tiller bud outgrowth [74]. Rice SL mutants were used to investigate whether SL elevated by Pi deficiency would inhibit tiller bud outgrowth. Under Pi deficiency, the levels of SL biosynthesis genes *D10*, *D17*, *D27*, and *OsMAX1* (*Os01g0700900*) transcripts in roots are elevated, and endogenous SL is increased, inhibiting the tiller bud outgrowth in wild-type seedlings, but tiller bud outgrowth was observed in *d* mutants [75]. Similar results were obtained in Arabidopsis [76]. Thus, SLs are thought to be signal molecules that mediate plant adaptation to Pi deficiency. In addition, SLs produced in roots are secreted into the soil and induce symbiotic interactions with arbuscular mycorrhizal fungi in the rhizosphere to acquire limited Pi. Therefore, SLs have an important dual role in efficient Pi utilization and acquisition under Pi deficiency [77]. Pea showed enhanced mycorrahizal colonization in a Pi-deficient condition compared to a Pi-sufficient condition. The enhanced colonization was also confirmed in *ccd8* mutants, indicating that SLs are not essential to show enhanced mycorrhizal colonaization under Pi deficiency [78].

To determine whether Pi deficiency would affect the SL responsiveness of leaf senescence in rice SL biosynthetic mutants, we examined the effect of exogenously applied GR24 on leaf senescence and found that GR24 responsiveness did not differ between Pi-sufficient and -deficient conditions in wild type and the *d14* mutant [44]. Generally, leaf chlorophyll content decreases under Pi deficiency [79,80]. However, chlorophyll levels increase in SL-biosynthesis mutants and *d3* under Pi deficiency [44]. As a result, stronger GR24 responsiveness was observed in SL-biosynthesis mutants under a Pi-deficient condition than a Pi-sufficient condition. Under Pi deficiency, Pi is redistributed from old leaves toward sink organs such as young leaves, growing roots, and developing seeds [81,82], which accelerates senescence of the old leaves. The SL signal is required for shoot branching inhibition and efficient progression of leaf senescence, suggesting that SLs might regulate phosphate allocation among plant tissues [83].

## 5. Nitrogen Metabolism and SLs

Nitrogen is required for the synthesis of nucleic acids, proteins, and secondary metabolites including nitrogen, and is a major limiting factor in plant productivity [73]. Under nitrogen deficiency, gene expression is repressed in amino acid synthesis, chlorophyll synthesis, photosynthetic light reaction, and Carvin cycle, and is induced in amino acid degradation. Levels of amino acids and nitrogen-containing compounds decrease, whereas starch and flavonoid levels increase. As mentioned above, SLs are produced in response to nitrogen deficiency in some species [67,70,71]. Plants use nitrate or ammonium ions (NH_4_^+^) as a primary nitrogen source. When NH_4_^+^ is assimilated in plant cells, glutamine synthase (GS) coupled with glutamate synthase (GOGAT) catalyzes the ATP-dependent addition of NH_4_^+^ to glutamate [84]. GOGAT generates two glutamate molecules from glutamine and 2-oxoglutalete in the presence of either ferredoxin or NADH. In rice, there are three cytosolic GS1 (*OsGS1;1*, *OsGS1;2*, and *OsGS1;3*) and two NADH-GOGATs (*OsNADH-GOGAT1* and *OsNADH-GOGAT2*) [85]. In chloroplasts, GS2 and ferredoxin-GOGAT are responsible for the assimilation of NH_4_^+^ released during photorespiration [85]. *OsGS1;2* and *OsNADH-GOGAT1* are important for primary NH_4_^+^ assimilation in the roots. Tiller bud outgrowth in rice is severely reduced by the lack of *OsGS1;2* [86]. Although a high SL content in rice roots is known to reduce the number of outgrowing tillers, the SL levels in *gs1;2* and *nadh-gogat1* are similar to those in wild type, indicating that the reduction of tiller bud outgrowth observed in *gs1;2* is independent of the SL level. On the other hand, *OsGS1;1* and *OsNADH-GOGAT2* were expressed in mature rice leaves [87]. *OsGS1;1* disruption causes a severe reduction of growth and grain filling [88]. Therefore, *OsGS1;1* and *OsNADH-GOGAT2* appear to be important in the remobilization of nitrogen during natural leaf senescence in rice. However, no information is available on the relationship between *GS1;1* and SLs during leaf senescence.

**Figure 3:**
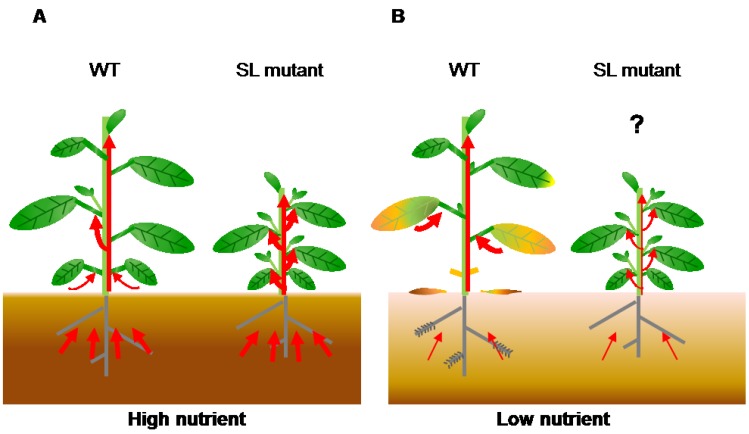
Transport of nutrients during leaf senescence under nutrient deficiency. (**A**) In nutrient-sufficient soil, plants absorb enough nutrients for growth and seed production. Wild-type plants show axillary bud outgrowth and the leaves remain green during leaf development because the SL levels are low. In SL mutants, the number of branches increases and nutrients absorbed from soil are evenly distributed between old and young organs because these plants have no SL signaling; (**B**) In nutrient-deficient soil, plants cannot absorb enough nutrients. In the wild type, increased SL levels inhibit shoot branching and accelerate leaf senescence to use limited nutrients. Nutrients are transported from old tissues to young tissues and seeds. SL mutants show enhanced shoot branching and delayed leaf senescence, which might reduce nutrient remobilization. Red arrows indicate flow of nutrients.

## 6. Perspectives

Plant growth is controlled by a complicated cross-talk of plant hormones. Recently, the interactions between SLs and other hormones have been uncovered in plant development [89] and stress responses [90,91]. However, the role of these interactions in leaf senescence is still unknown. Our hypothesis is that plants grown in soil with sufficient concentrations of nutrients (such as nitrogen and Pi) receive enough nutrients from the roots, and axillary buds can grow because SL levels are low (Figure 3). In contrast, plants grown in nutrient-deficient soil produce high SL levels to inhibit axillary bud outgrowth and accelerate leaf senescence for utilization of limited nutrients (Figure 3). To make up for nutrient deficiency, nutrients accumulated in old leaves are transported to young tissues and seeds. Because SL mutants show enhanced shoot branching and delayed leaf senescence, they may have reduced nutrient remobilization, resulting in a reduction of seed production. These findings indicate that SLs might affect crop grain yield through leaf senescence and shoot branching regulation. To address this question, it is necessary to compare the levels of primary metabolites (such as sugars and amino acids) and seed production in wild-type plants and SL mutants in the future.
